# Supplementary material for: Unique N-glycosylation signatures in human iPSC derived microglia activated by Aβ oligomer and lipopolysaccharide
Source: Sci Rep. 2025 Apr 10;15:12348. doi: 10.1038/s41598-025-96596-1 (PMC11985925; doi:10.1038/s41598-025-96596-1)
Supplement: Supplementary file 3 — Supplementary Material 3 [file 41598_2025_96596_MOESM3_ESM.docx]

**Title: Unique N-glycosylation signatures in human iPSC derived microglia activated by Aβ oligomer and lipopolysaccharide**

Running title: AβO and LPS induce differential glycosylation changes in microglia

Xinyu Tang^1^*, Ryan Lee Schindler^2^*, Jacopo Di Lucente^3^, Armin Oloumi^2^, Jennyfer Tena^2^, Danielle Harvey^4^, Carlito B. Lebrilla^2^, Angela M. Zivkovic^1^**, Lee-Way Jin^3^**, Izumi Maezawa^3^**

^1^Department of Nutrition, University of California, Davis, California, USA.

^2^Department of Chemistry, University of California, Davis, California, USA.

^3^Department of Pathology and Laboratory Medicine and M.I.N.D. Institute, University of California Davis Medical Center, Sacramento, California, USA.
^4^Department of Public Health Sciences, University of California-Davis, Davis, CA, USA.

*Authors have equal contribution to the manuscript.

**Address Correspondence to:** Izumi Maezawa, Ph.D., Department of Pathology and Laboratory Medicine, University of California Davis Medical Center, Sacramento, CA 95817, USA, E-mail: [imaezawa@ucdavis.edu](mailto:imaezawa@ucdavis.edu), Lee-Way Jin, MD., PhD., Department of Pathology and Laboratory Medicine, University of California Davis Medical Center, Sacramento, CA 95817, USA, E-mail: [lwjin@ucdavis.edu](mailto:lwjin@ucdavis.edu), Angela M. Zivkovic^,^ PhD., Department of Nutrition, University of California, Davis, CA 95618, USA, E-mail: [amzivkovic@ucdavis.edu](mailto:amzivkovic@ucdavis.edu).

Table of Contents

[Supplementary figure 1 2](#_Toc193746388)

[Supplementary figure 2 2](#_Toc193746389)

[Supplementary figure 3 3](#_Toc193746390)

[Supplementary figure 4 4](#_Toc193746391)

[Supplementary table S1 The relative abundance of N-glycans 4](#_Toc193746392)

[Supplementary table S2 The absolute abundance of GSLs 4](#_Toc193746393)

# **Supplementary figure 1**


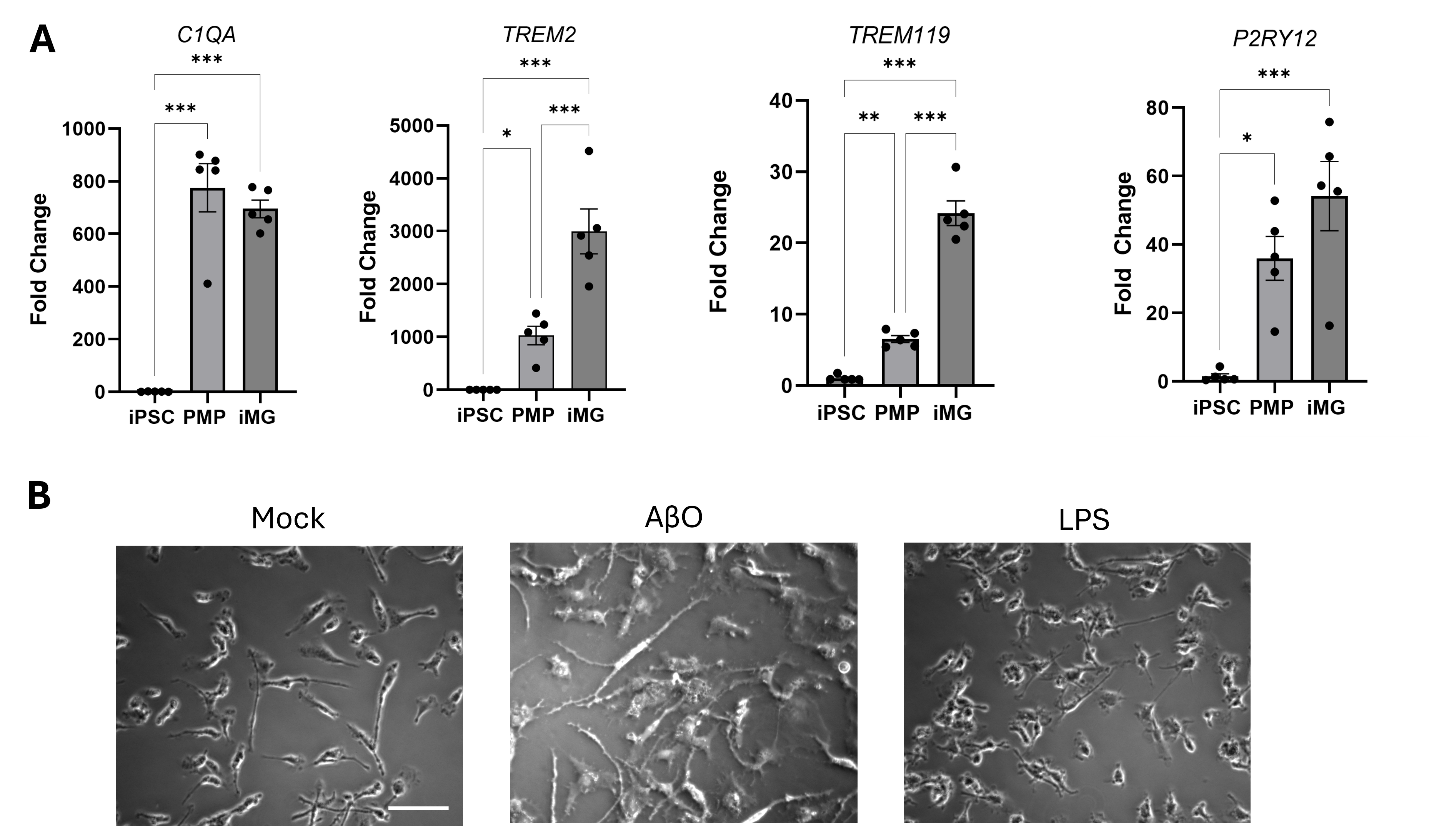


**Supplementary figure 1.** Verification of mature microglia-like cells differentiated from iPS cells. A) RNA was isolated from parent iPS cells (iPSC), primitive macrophage precursor (PMP) and mature microglia (iMG). Mature microglia markers were quantified by qPCR. B) iMG were treated with mock treatment, AβO (3 μM), or LPS (100 ng/ml) for 24 hrs before phase contrast images were taken. Scale bar :50 μm. Compared to mock treatment, both AβO- and LPS-treated iMG showed more cells with hypertrophic and ameboid morphology.

# **Supplementary figure 2**


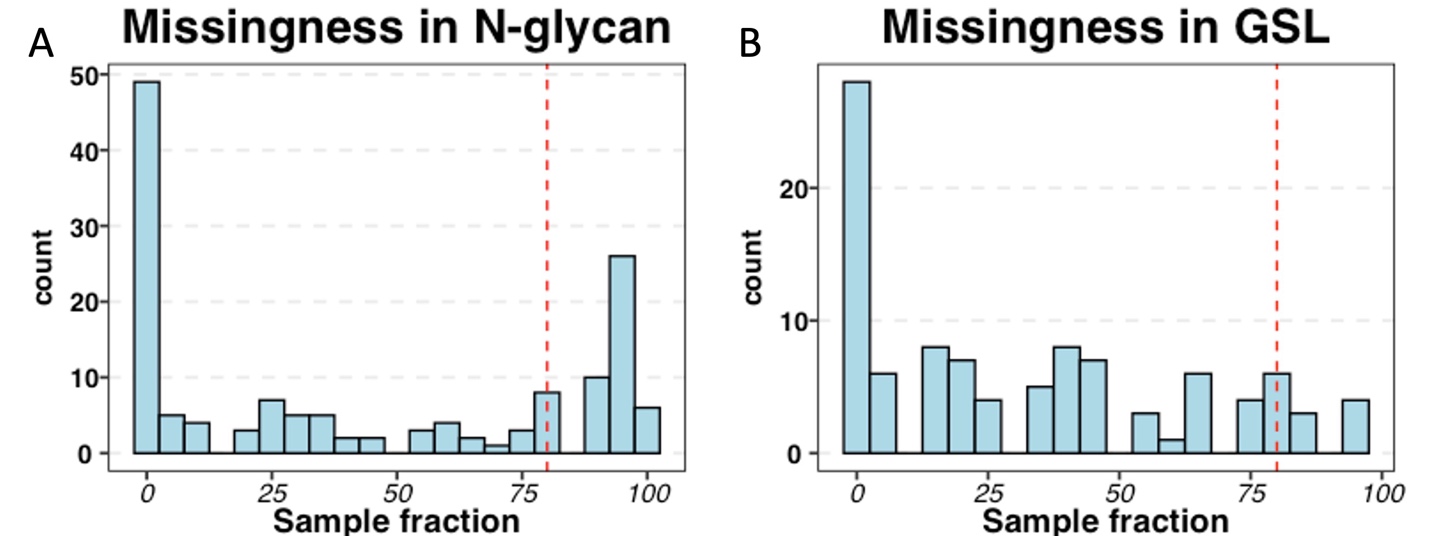


**Supplementary figure 2.** The missingness of N-glycans and GSL as the percentage of the number of samples. A) The missingness in N-glycan. B) The missingness in GSL. Red dash lines denote missingness of 80% across all samples.

# **Supplementary figure 3**


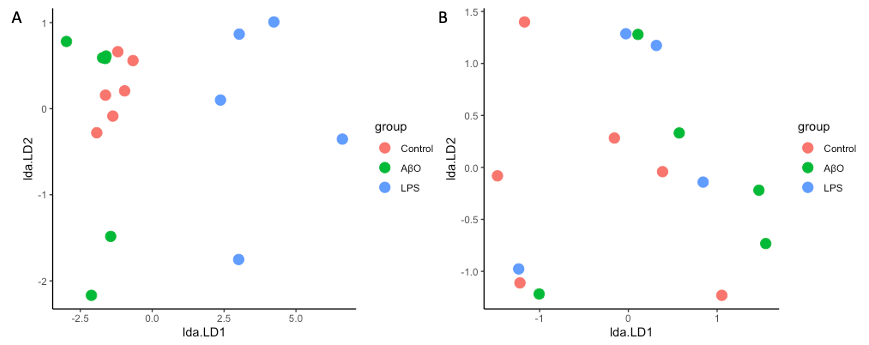


**Supplementary figure 3**: The scatter plot of the post-hoc linear discriminant analysis (LDA) of MANOVA for the PCA plots. A) The LDA scatter plot for N-glycan. According to the LDA scatter plot, LPS had the most significant impact on the group mean differences. B) The LDA scatter plot for GSL.

# **Supplementary figure 4**


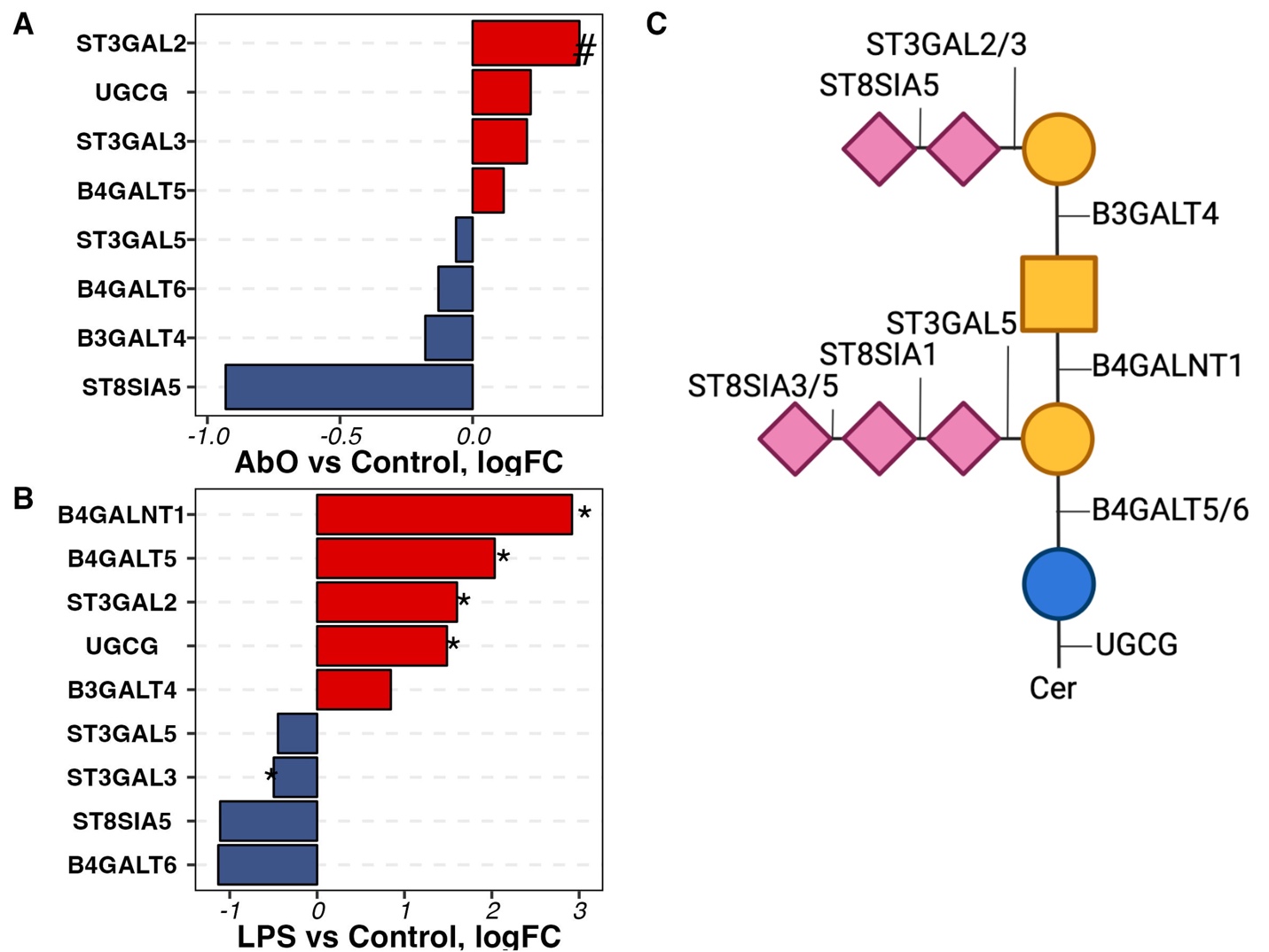


**Supplementary figure 4.** The differential expression of genes involved in GSL biosynthesis in AβO- and LPS-activated hiMG. A) The differential expression of genes involved in GSL biosynthesis in AβO-activated hiMG. B) The differential expression of genes involved in GSL biosynthesis in LPS-activated hiMG. C) The schematics of GSL structure and the corresponding enzymes. The pound (#) denotes genes with unadjusted p-value < 0.05 but FDP >0.05. The asterisk (*) denotes genes with FDP < 0.05.

# **Supplementary table S1 The relative abundance of N-glycans**

See the separate .csv file

# **Supplementary table S2 The absolute abundance of GSLs**

See the separate .csv file
